# Large Clitoral Leiomyoma in a Forty-Two Years Old Premenopausal Woman

**DOI:** 10.5812/numonthly.17022

**Published:** 2014-05-12

**Authors:** Santosh Kumar, Swati Agrawal, Kumar Jayant, Sriharsha Ajjoor Shankargowda

**Affiliations:** 1 Department of Urology, Institute of Medical Education and Research, Chandigarh, India; 2 Department of Urology, Medical College, Udaipur, India

**Keywords:** Leiomyoma, Clitoral, Premenopausal

## Abstract

**Introduction:**

Clitromegaly can be congenital or acquired with the former type being more prevalent. The main etiology behind the acquired type is hormonal imbalance affecting mainly adult women. This type is seen mostly in association with polycystic ovarian syndrome, hyperthecosis, ovarian tumors, and clitoral cysts or it can be drug-induced. Clitoral leiomyoma is a rare benign tumor of female genitalia and is an uncommon cause of clitromegaly.

**Case Report:**

We reported a 42-year-old premenopausal woman with a progressively increasing mass since five years ago, who had attended our clinic with urinary retention. It was a fungating mass with ulceration arising from the crus of the clitoris with the size was 11 × 9 cm. After detailed laboratory investigations, she had normal karyotyping. Hormonal assay for testosterone, dehydroepiandrosterone sulphate, and follicle stimulating hormone, luteinizing hormone, parathormone, and prolactin levels revealed values within the normal range. Twenty-four hours urinary excretion levels of free cortisol and ketosteroids were within normal limits. Beta-hCG level was also in normal range. Thyroid function tests and X-ray chest results were normal. Contrast enhanced computed tomography (CECT) of the abdomen showed no abnormality in adrenals and there was no pituitary enlargement on brain MRI. Pelvis MRI showed a large 11 × 9 × 8 cm clitoral mass. Diagnostic biopsy done from ulcer margin was suggestive of leiomyoma. The mass was completely excised preserving the tip of clitoris. The histopathology showed spindle-shaped cells arranged in palisading form. On immunohistochemistry, tumor cells were positive for smooth muscle actin (SMA) as well as for estrogen and progesterone receptor (ER/PR), confirming the diagnosis of leiomyoma. The patient was regularly followed, and was doing well with no voiding difficulty.

**Conclusions:**

We reported the world largest clitoral leiomyoma presenting with symptoms of acute urinary retention. MRI has important role in diagnosis and biopsy is confirmative with spindle-shaped cells arranged in palisading pattern and simple excision would be curative.

## 1. Introduction

Clitromegaly is defined as an unusual increase in the size of clitoris, which may be congenital or be acquired. In comparison to congenital types, acquired types are rare. the acquired types are commonly seen in association with polycystic ovarian syndrome (PCOS), cystic lesions, hyperthecosis, and neurofibromatosis or they can be related to intake of certain drugs such as Danazol (1). Herein, we reported the largest clitoral leiomyoma presenting with symptoms of acute urinary retention that was not reported in the literature so far.

## 2. Case Report

**Figure 1.**
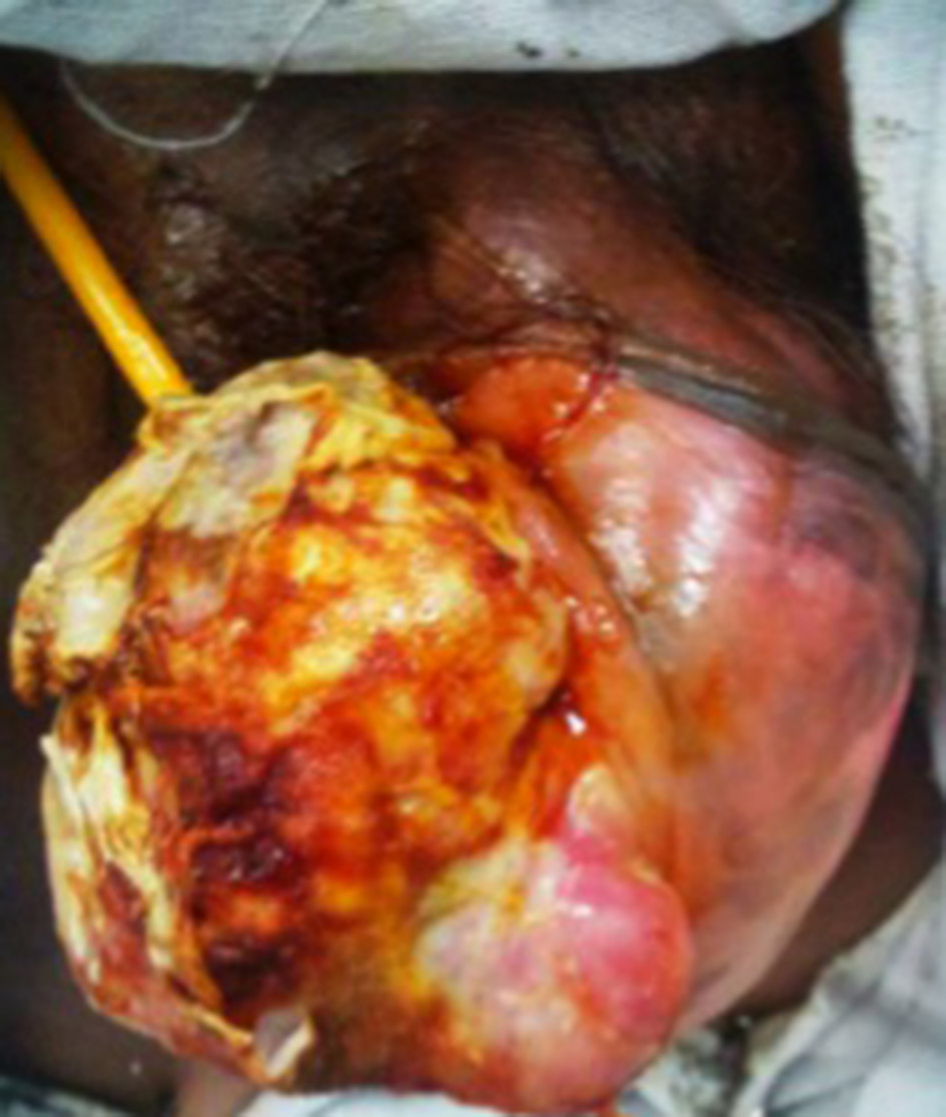
Large Fungating Clitoral Leiomyoma With Placed Urethral Catheter

**Figure 2.**
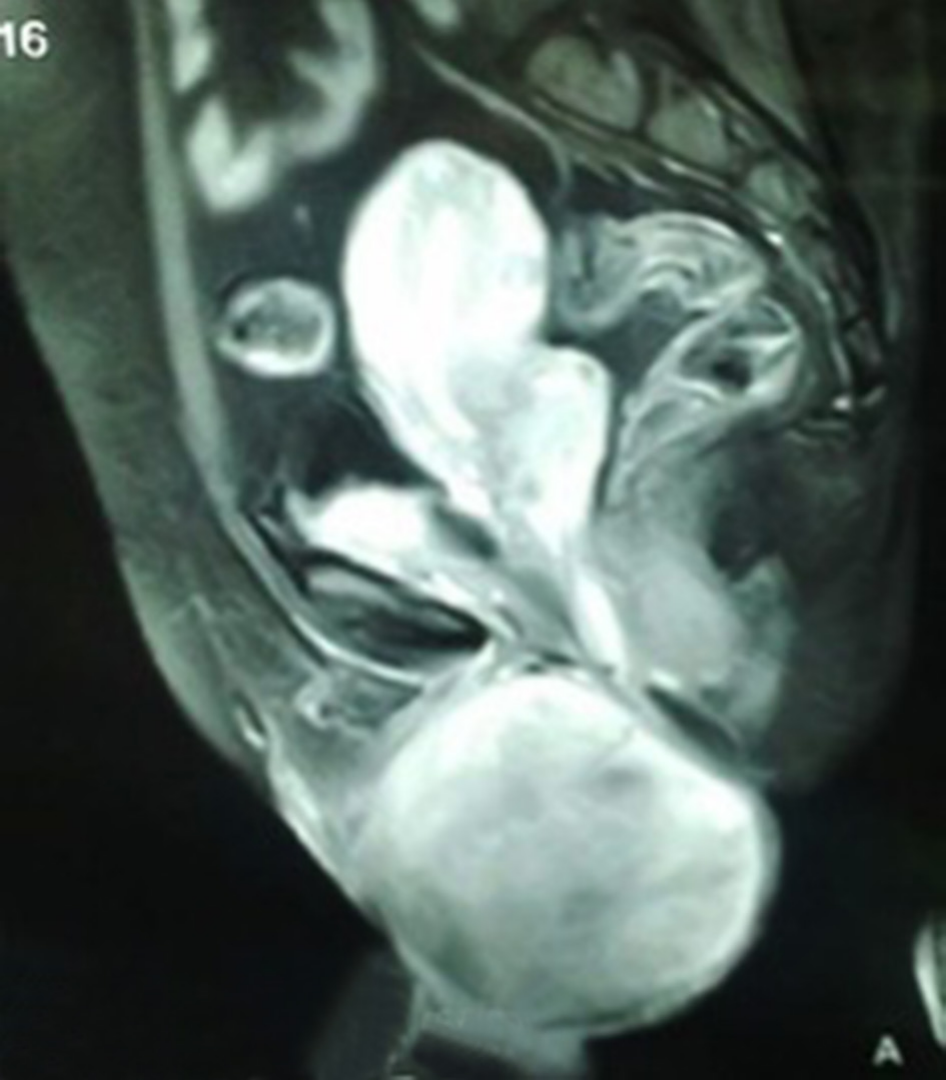
Magnetic Resonance Imaging (T2-Weighted, Sagittal Fat Suppression Image) Showing a Larger Mass Arising from Clitoris With Compression of the Urethra

**Figure 3.**
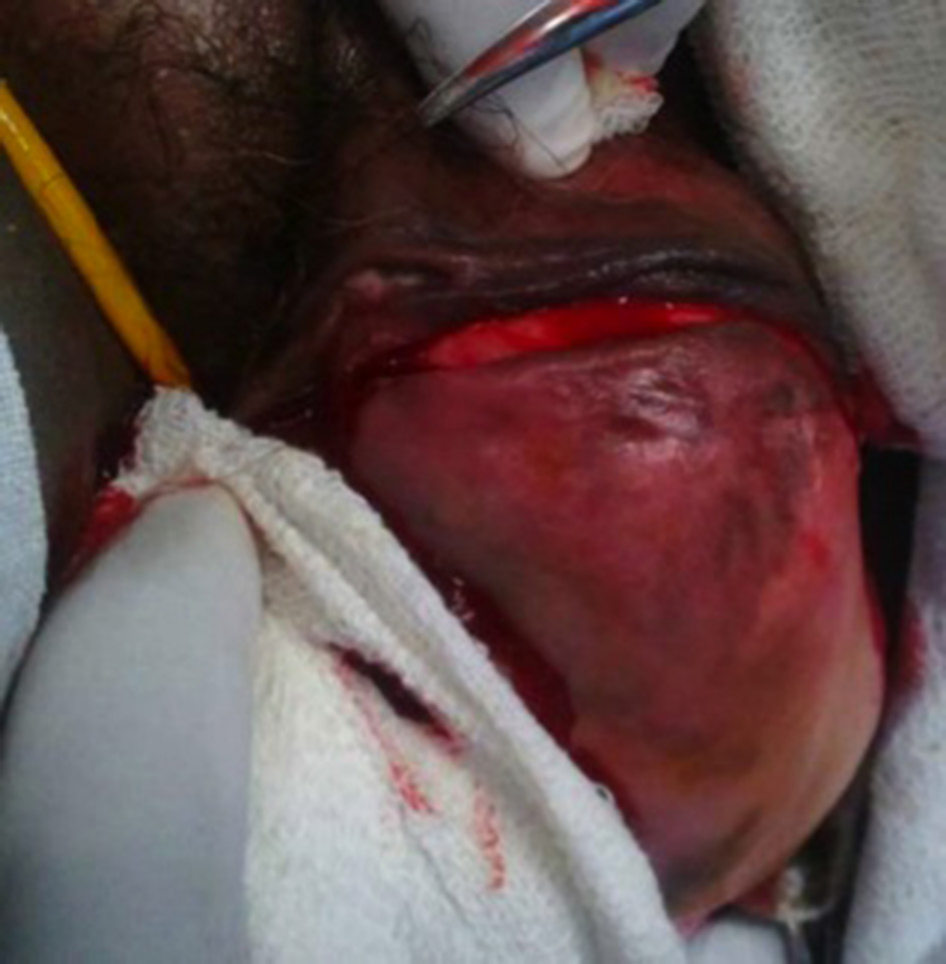
Intraoperative Photograph Showing Incision Over the Clitoral Mass With Preservation of the Tip of Clitoris

**Figure 4.**
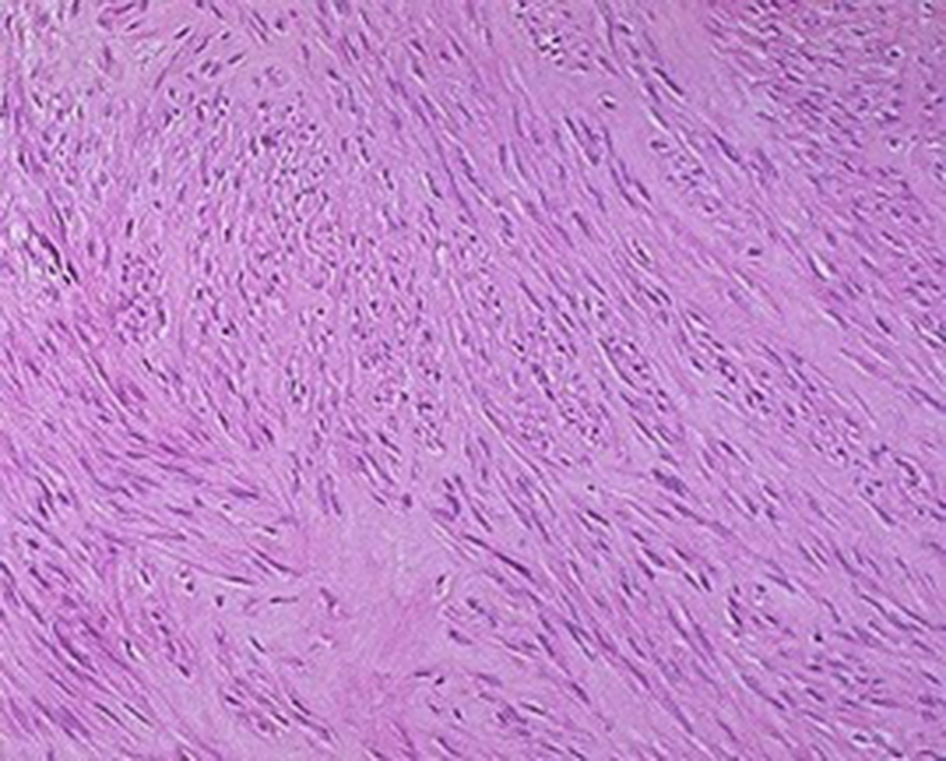
Microscopic Picture Showing Spindle-Shaped Tumor Cells Arranged in Palisading (Hematoxylin-Eosin Stain, Magnification × 400)

A 42-year-old premenopausal woman attended our emergency department with acute urinary retention. She also had complaint of swelling in the vulval region since five years ago, which was painless and gradually progressive. On local examination, there was an 11 × 9 cm fungating, nontender, solid mass arising from the clitoris (Figure 1). Pelvis magnetic resonance imaging (MRI) was requested at the former hospital where she had gone for treatment of her vulval mass; the imaging revealed a large 11 × 9 × 8 cm clitoral mass with a characteristic finding of low signal intensity on T2-weighted images mimicking that of smooth muscle, which was the key to the diagnosis (Figure 2). Patient was initially managed in the emergency department with urethral catheterization relieving urine retention. On laboratory evaluation, her hemoglobin was 10 g/dL with normal renal function tests (serum creatinine, 0.9 mg/dL; urea, 21 mg/dL). On abdomen ultrasound evaluation, bilateral kidneys were normal. Hormonal assay showed normal values of testosterone, dehydroepiandrosterone sulphate, follicular stimulating hormone (FSH), luteinizing hormone (LH), parathormone (PTH), and prolactin. The 24-hour urinary excretion of free cortisol and ketosteroids were within normal limits. Beta-hCG level was also in normal range. Thyroid function tests and chest X-ray findings were insignificant. Contrast enhanced computed tomography (CECT) of the abdomen showed normal adrenals. There was no pituitary enlargement on brain MRI. Afterwards, decision for surgery was made and the mass was excised completely with preserving the tip of clitoris. Resected tumor mass was sent for histopathological assessment (Figure 3). Postoperative course was uneventful with no voiding difficulty. On gross examination, the mass was well encapsulated and section cuts showed fleshy tumor. Microscopic examination showed spindle-shaped tumor cells arranged in fascicles with few areas of myxoid degeneration; moreover, hyalinization was seen without any atypia or mitotic figures (Figure 4). Immunohistochemistry (IHC) was positive for smooth muscle antigen (SMA) and estrogen and progesterone receptor (ER/PR). Overall features were suggestive of clitoral leiomyoma. Our regular follow-up showed that the patient was doing well without any recurrence of lesion or any difficulty in voiding.

## 3. Discussion

Uterine leiomyomas are the most common benign tumors of female genital tract in females at reproductive age. Majority of leiomyomas arise in the reproductive period and resolve after menopause. Although the exact etiology is not known, one of the main pathogenesis is estrogen- and progesterone-induced tumor growth. According to this theory, increased levels of estrogen and progesterone result in an increased mitosis of cells leading to myoma formation, which is associated with high likelihood of somatic mutations (2). Clitoral leiomyoma is a rare benign tumor. Based on the size, clitromegaly is defined as clitoris larger than 35 mm^2^, which is almost twice the normal size. The origin of the clitoral leiomyoma, unlike the vaginal leiomyoma, is not clearly determined and it is not related to mullerian tract. Although it is a matter of dispute, few studies suggested the surrounding mesenchyme of the urogenital sinus as a presumptive origin of clitoral leiomyoma (3).

The clitoral leiomyomas are usually asymptomatic, painless, and non-tender but can present with dysuria, urinary frequency, urinary retention, and dyspareunia (4). MRI is useful not only in diagnosis but also in differentiation of benign from malignant tumor. A characteristic finding is of low intensity signals that resembles that of smooth muscle on T2-weighted images and isointense to that of muscle on T1-weighted images with homogeneous enhancement on intravenous contrast-enhanced images. Diagnosis is confirmed with biopsy, which shows spindle-shaped tumors cells arranged in palisading pattern with few areas of myxoid and hyaline degeneration without any atypia or mitotic figures. IHC studies show positivity for SMA and ER/PR (5-7). These tumors must be considered malignant if they have three or more of the following criteria (8, 9):

1. Five centimeter or more in size in their maximum diameter
2. Infiltrative margins
3. Five or more mitotic figures per ten high power fields
4. Moderate to severe cytological atypia

Hereby, we presented the largest clitoral leiomyoma documented in the literature. It was a benign tumor since it did not fulfill the rest of criteria of malignancy such as infiltrative margin, presence of atypia, or mitotic figures. Surgery in the form of simple excision is usually sufficient. Because of estrogen receptor expression in the tumor, the conservative treatment with long acting gonadotropin releasing hormone (GnRH) agonists or antagonists may be used as an adjuvant therapy (10). MRI has important role in diagnosis and biopsy is confirmative with spindle-shaped cells arranged in palisading pattern. Simple excision is curative.
